# Experiences of dementia in a foreign country: qualitative content analysis of interviews with people with dementia

**DOI:** 10.1186/1471-2458-14-794

**Published:** 2014-08-04

**Authors:** Monir Mazaheri, Lars E Eriksson, Alireza Nikbakht Nasrabadi, Helena Sunvisson, Kristiina Heikkilä

**Affiliations:** School of Health, Care and Social Welfare, Mälardalen University, Box 325, 631 05 Eskilstuna, Sweden; Department of Neurobiology, Care Science and Society, Karolinska Institutet, Stockholm, Sweden; Faculty of Nursing and Midwifery, Tehran University of Medical Sciences, Tehran, Iran; Department of Infectious Diseases, Karolinska University Hospital, Stockholm, Sweden; School of Health Sciences, City University London, London, United Kingdom; School of Health and Medical Sciences, Örebro University, Stockholm, Sweden; Department of Health and Care Sciences, Faculty of Life and Health Sciences, Linnaeus University, Kalmar, Sweden

**Keywords:** Dementia, Experience, Qualitative studies, Semi-structured interview, Immigration, Iran, Sweden, Content analysis

## Abstract

**Background:**

Dementia is a worldwide health concern of epidemic proportions. Research in the field of subjective experience of dementia suffers from a lack of diversity of their participants including immigrants. Different portraits of life with dementia could help us understand how people with dementia conceptualise their experiences of dementia and how they live. Our study aimed to explore the subjective experiences of living with dementia among Iranian immigrants in Sweden.

**Methods:**

Qualitative content analysis of interviews with fifteen people with dementia from Iranian immigrant backgrounds were conducted (8 females and 7 males).

**Results:**

Three themes and seven associated sub-themes were revealed. The themes included: Being a person with dementia means living with forgetfulness (personal sphere), living with forgetfulness in the private sphere means feeling incompetent but still loved, living with forgetfulness in the public sphere means feeling confident and secure but also isolated.

**Conclusions:**

Living with dementia for the participants meant living with forgetfulness. They experienced feeling incompetent but still loved within their families and feeling confident and secure but also isolated in the society. Educating people with dementia and their families about the course and process of dementia may help them understand the changes better and adjust their expectations. Our study can provide a basis for healthcare workers to understand the experiences of living with dementia from this specific perspective.

## Background

Dementia is a worldwide health concern of epidemic proportions [1] and a serious health challenge that decreases older people’s chances of living independently [2]. The way the various forms of dementia affect brain functions—for example, language ability—is universal but people’s behaviors, reactions, and responses to dementia are different for various reasons, including their personal history and cultural background [3].

The experience of living with dementia has been described mainly to have negative consequences [4], including a variety of losses for people with dementia [5], as well as burden [6], strain, stress [7], poor physical and mental health [8] and low quality of life [9] for their families. However, most research on dementia from the perspective of people with dementia has been conducted on mainstream populations in western countries. Furthermore, in many studies, immigrants are grouped together with other minority ethnic groups, which makes it difficult to study the potential impact of the experience of immigration on the experiences of people with dementia.

Although the body of knowledge is not very large and there is a low grade of diversity of the participants [10], there are some studies addressing experiences of dementia in other countries and among ethnic groups and immigrant populations in western societies. These studies have revealed different cultural constructions of dementia, from a normative understanding in India [11] to a ‘supernormal’ construct of the disease in native Americans where dementia-related symptoms are perceived as normal while the person is perceived as being in touch with a supernatural world [12].

Differences in knowledge of dementia have also been found among different ethnic groups; for example, in California, Asian, African-American and Hispanic people were found to have less knowledge about Alzheimer’s than Caucasians [13]. Moreover, language insufficiencies among immigrant seniors have been suggested to lead to isolation [14]. Even bilingual people with dementia have been found to lose their secondary language abilities, causing problems in social situations according to a Swedish study focusing on Finnish immigrants [15]. From a Swedish perspective, studies that address the subjective experience of living with dementia from the perspective of people with immigrant backgrounds in the country are scarce. One study [16] has reported difficulties in recruiting immigrants in Sweden to dementia research for reasons such as their lack of knowledge about dementia and Swedish rules and regulations, sense of insecurity, and cultural view of dementia.

While immigrants and ethnic minorities constitute nine percent of Europe’s population [17], their voices have not been well represented in dementia research. In Sweden, almost 13% of the population over 65 years old is of foreign origin [18] and the presence of an aging immigrant population raises important concerns about the well-being of this older people population. Iranian immigrants who moved to Sweden, the great majority of whom did so in the years after the Islamic revolution in Iran in 1978, are the second largest non-European community in Sweden (n = 81190) [19] and are now ageing. The aim of the present study was to explore the subjective experience of living with dementia among Iranian immigrants in Sweden. Different portraits of life with dementia can help us understand how people with dementia conceptualise their experiences of dementia and how they live. This knowledge is a prerequisite for tailoring care systems for people with dementia and their families.

## Methods

### Design

A descriptive qualitative design [20], based on individual interviews with people of Iranian origin who had immigrated to and now were living with dementia in Sweden, was chosen for the present study. The RATS guidelines were followed in reporting the present study.

### Study context

The study was conducted in the three large cities of Stockholm, Uppsala, and Lund in Sweden, which has become a multicultural country because of immigration [21].

Sweden has a tax-financed health system and municipalities are responsible for providing general access to health care (Wright & Henry, 2012). People living with dementia in Sweden are cared for at home for as long as possible. Special housing is offered to those older people who are not able to fend for themselves.

### Participants

Fifteen people with Iranian immigrant backgrounds living with dementia in Sweden were recruited to the study purposefully. The first author contacted care centres for people with dementia to reach potential participants. The main inclusion criteria were that the candidates had been diagnosed as having dementia with Alzheimer’s disease or vascular dementia at least one year prior to the interview, had the ability to talk about their experiences, and were born in Iran and lived in Sweden. Staff at the care centres made the first approach to potential participants and their families to ask if they would like to participate in the study. The first author then contacted each participant directly by phone to give them more information about the study and, if consent was obtained, to schedule an appointment for conducting the interview.

The participants included 15 people (8 females and 7 males) with dementia, ranging in age from 66 to 88 years. Ten of them were living at home, either alone (6 people) or with their family (2 with spouses and 2 with daughters), and the remaining five were living in group dwellings for people with dementia. All had immigrated to Sweden 10 to 30 years before the interview.

### Data collection

Semi-structured interviews were conducted in Persian to elicit the participants’ experiences of living with dementia. The interviews took place in the participants’ homes or care centres, according to their wishes. The interviewees were asked to narrate their experiences of living with dementia. The interviews commonly started with an opening question (‘Please tell me about your experience of dementia’), which was probed with more specific questions to get clarifications, more details, and concrete examples of what they wanted to share with the interviewer (MM). The interviews were audio-recorded, transcribed verbatim, and validated by re-listening to the audio-tapes.

### Data analysis

The interviews were analysed according to qualitative content analysis [20] inductively. Qualitative content analysis, as a widely used qualitative research technique [22], has been applied in various materials, especially for coherent analysis of interview data in care science research and education [20]. The method is recommended for use when there is not enough knowledge about the subject or if the knowledge is fragmented [23], as is the case for the experience of living with dementia from the perspective of Iranians living in Sweden.

The transcribed interviews were first read through to acquire an overall understanding of the content related to the research aim. Then, the data were read word-for-word to derive meaning units. To achieve immersion and obtain a sense of the whole, all data were read repeatedly and discussed among the authors. The process continued to distill condensed meaning units and construct sub-themes and themes [20].

The two Persian-speaking authors (MM and ANN) conducted the analysis simultaneously, and two full interviews in English were provided for the non-Persian-speaking researchers, together with translated selections of each of the other interviews. The entire data were discussed in many research sessions with all authors, which included reading and going back to the original data, clarifying the issues that were not clear in the translated quotations, and further oral translations of additional parts when needed. All authors were involved in the process of constructing sub-themes and themes. The trustworthiness criteria of Lincoln and Guba (1985) were carefully followed to ensure the study’s rigor.

### Ethical considerations

The study was approved by the Regional Ethical Review Board in Stockholm (111/05). Information (available in Persian, Swedish, and English) about the study, including the aim of the study, the voluntary nature of participation, and the opportunity to withdraw at any time without cause or notice, was provided to the participants and their family members orally and in writing. None of researchers were involved in care management of participants or working with the healthcare settings that supported the participants. All participants gave their consent orally, which was recorded along with the interview. Iranian immigrants have been reported to feel insecure and suspicious of other Iranians [24], so we intentionally did not ask for their written consent. During data collection, every possible effort was made to detect non-verbal signs of inconvenience or indications of wishes to withdraw from the interviews.

## Results

The participants were capable of understanding and responding to questions and giving concrete examples of their experiences of living with dementia. They recalled conversations with relatives and family members in relation to the interview questions, thereby demonstrating their ability to explain and describe their experiences.

The content analysis revealed three themes and seven associated sub-themes (see Table 1), which reflect the participants’ experiences of living with dementia in three spheres: the person her- or himself (personal sphere), in the family sphere (private sphere), and in society (public sphere). The themes include: Being a person with dementia means living with forgetfulness (personal sphere); Living with forgetfulness in the private sphere means feeling incompetent but still loved; and Living with forgetfulness in the public sphere means feeling confident and secure but also isolated.

**Table 1:** **Themes and subthemes revealed by qualitative content analysis of interviews with Iranian immigrant with dementia in Sweden**

Themes	Subthemes
Being a person with dementia means living with forgetfulness (personal sphere)	Understanding of the forgetfulness
	Coping with the consequences of forgetfulness
Living with forgetfulness in the private sphere means feeling incompetent but still loved	Feeling incompetent in family roles
	Feeling loved and respected
Living with forgetfulness in the public sphere means feeling confident and secure but also isolated	Feeling secure
	Feeling confident in interacting with society
	Feeling isolated

### Being a person with dementia means living with forgetfulness (personal sphere)

When they were discussing their condition and the consequences of living with dementia, the participants mainly described dementia as forgetfulness. For them, forgetfulness was the main character of the disease of dementia. A 75-year-old woman expressed it in this way: ‘Dementia just means forgetfulness. Just that, I put things here. Then I just forget it. Yes.’ The participants attributed the difficulties of living with dementia to the problems arising from their forgetfulness. The participants also narrated their perception of dementia, how they feel about forgetfulness, its causes, and the ways they try to cope with it.

Different feelings accompanied their experiences of forgetfulness, like being afraid of forgetting things and what the consequences could be.

‘I myself get upset when I misplace things, which makes them [her family members] search a lot to find them…I want the house to be neat, clean, and well-organized. So I get angry.’ (75-year-old female)

-  
*Understanding of the forgetfulness*

The participants strove to understand why they had acquired their forgetfulness and how their condition had developed. They had different understandings of and suggestions for the causes of it. They perceived a wide range of circumstances that had contributed in developing their forgetfulness, such as, for instance, having had a hard life, or having experienced traumatic events.

‘… My problem started from that point. When he got his second wife, I had such a strange strong feeling that I forgot everything.’ (83-year-old female)

Some also perceived the forgetfulness to be an age-related weakness that was natural due to body changes during the ageing process.

When describing life with dementia, some participants seemed to distance themselves from the disease by describing other people with dementia when they discussed the probable causes for getting dementia. This was evident especially when they considered how other people had gotten dementia. For example, they thought that getting the disease was related to low education or intelligence quotient (IQ) or a punishment for earlier acts in life. There was one person who regarded dementia as a disease caused by God’s punishment:

‘When I see people with dementia, I say to myself, they are psychiatric patients. They are mentally ill. I say to myself, ‘why God, what had they done in their youth to have such ending? How have they lived to find themselves in such a condition of forgetfulness?’ (83-year-old female)

However, their own problems of living with forgetfulness and its consequences were not perceived as connected to a low education level or low IQ and were not felt to be a punishment for earlier acts or from God.

-  
*Coping with the consequences of forgetfulness*

To deal with problems induced by forgetfulness, different strategies were used. Participants tried to normalise, rationalise, or downplay the extent of their forgetfulness. An example of normalising the forgetfulness was a 66-year-old female who believed that it was normal for people of her age and condition to experience forgetfulness and that there was therefore no real problem:

‘I want to say something. I forget what I wanted to say. [Laughing.] You know we are not 14 years old anymore. [Laughing.]…I calculated money wrongly. But no worries. I pay 5 SEK [Swedish currency] less, they say it’s wrong. Then I pay 5 SEK more.’ (66-year-old female)

Most of the participants tried to downplay their forgetfulness, believing they still had much in life to be happy with, things that living with forgetfulness could not take away. A 66-year-old female participant mentioned that ‘I don’t do anything important that makes me worried about that if I forget, there would be serious consequences. So it is fine with my forgetfulness’. She and some of the other participants believed that dementia did not have much impact on their lives since they were not working or doing some vital activities which could harm someone, so they did not perceive the forgetfulness as a problem.

Some even mentioned they benefitted from their forgetfulness and tried to rationalise it in that way. A 72-year-old male said that he even takes advantages of his forgetfulness:

‘I should be honest with you. When I see there are too many inquiries, questions, and so on … if I want to divide my energy for all of them, nothing would be left for me. So it’s better to just forget those things that are not important that much … my wife asks me to go to buy something special. I go out but since my financial status is not too good at the time and I can’t afford the costs, I forget the shopping list, on purpose! [Laughing] You know, I pretend to forget. When they ask me about their requests, I say, oh, my God, see! I just forgot that! You know, if you forget something, they don’t execute you for your forgetfulness!’

To prevent the progress of forgetfulness or avoid its consequences, participants used different strategies, like exercising their memory skills, concealing their inability to remember or respond satisfactorily, and comparing themselves with those who had worse situations.

‘I call someone to ask them to bring something to me. When they come, I have forgotten what I wanted. Then I just greet and thank them, without saying I forgot what I wanted.’ (74-year-old male)

### Living with forgetfulness in the private sphere means feeling incompetent but still loved

Participants discussed how forgetfulness raised problems in their family lives. They expressed their feelings of incompetence in meeting their family members’ and relatives’ expectations. Although their family members blamed them for behaviours that caused the family members trouble, the participants still felt loved.

-  
*Feeling incompetent in family roles*

Forgetfulness, as an apparent problem of dementia, disturbed family functions. Participants had experienced family conflicts, for example, because they were suspicious of family members and mistrusted people close to them regarding lost items whose location they had forgotten and that they found later.

Some of the participants found themselves embarrassed in situations where some of their actions had led to difficulties for their families or their friends. They tried to behave socially appropriately to please their children and family. The reason was to show that they still were worthy of being valued. They became ashamed that they lost their competence and blamed themselves for the practical consequences of their conditions like misplacing household items and not being able to locate them when others needed them.

‘My daughter put a paper somewhere. As I wanted it not to get lost, I took it and put somewhere better place. Later, she said to me why did I take her stuff and misplace it. I told her to search around to find it. She said, “No, I can’t, I have a lot to do, why did you take it?”…I get embarrassed when they are not able to find things. I regret that I took those things. But, you know, I cannot stop myself organizing my surroundings.’ (75-year-old female)

They also mentioned difficulties remembering the steps of some tasks, maintaining conversations with their relatives, doing daily chores, being confused about the surrounding environment, and being stressed about their probable mistakes.

Some of the participants felt incapable when they were not able to act upon their relatives’ requests or upon their own wishes. Needing others’ help for moving around or providing what they needed was mentioned as one of the contributors to feeling incapable compared to their previous conditions. They were not being allocated responsibilities at home and they were aware of how their families discussed their incapability of doing things.

‘You know my children know about my forgetfulness. My daughter tells my son not to give responsibilities to mom or expect much. She forgets things…’ (83-year-old female)

-  
*Feeling loved and respected*

Regardless of their problems, several participants felt loved and respected. Some also thought that because of their relationship and seniority, they would continue to be respected, whether or not that respect was based on their relative’s interest in them or was only a cultural practice considering their age and relationship with the family members.

‘Other people like your children wouldn’t say anything. If it [the person with dementia] is your mother, it’s your father who has forgotten something, what do they want to say? Nothing! They don’t say anything….if it’s your father, your mother, what do you want to say? [They wouldn’t complain.]’ (70-year-old male)

### Living with forgetfulness in the public sphere means feeling confident and secure but also isolated

Participants described how they lived with dementia in society. They felt fine and secure in going around and doing activities in their surrounding community even though they mentioned events where their ability to manage had been compromised, even if they had managed to overcome the events in one way or another. However, some also perceived isolation since they were not able to communicate in Swedish.

-  
*Feeling secure*

In general, most participants felt comfortable. They felt themselves secure in their general condition despite dementia and its consequences.

‘Here [in Sweden] is very good. You have your own place, you have money. Why should you worry then… you know here, I can go out whenever I want.’ (69-year-old female)

Nevertheless, they had been faced with many problematic situations. Several participants described how they had experienced problems like getting lost but being safely returned home. In these cases, as the situations had ended positively from their point of view, they felt safe and such experiences didn’t scare them into changing their activities like outdoor wanderings. For example, although commuter trains in Sweden are not free of charge, a 66-year-old female said:

‘I take the train. The trains are free. I take it. I say ‘tack, tack’ [thanks, thanks]. See, it is very easy. Here is easy to go around. They are very nice to me. I even take my son, say ‘kom, kom’ [come, come]. I take him in the train too. Very good!’ (66-year-old female)

-  
*Feeling confident in interacting with society*

In general, most of the participants continued to be pleased with being able to interact with their surrounding society. They were not afraid of going out to do activities like visiting a graveyard or taking a walk alone in the community. They gave many examples of how their problems due to dementia—like disorientation with respect to place, directions, misunderstandings, and their forgetfulness—had been handled safely and calmly with the help of police or other people. They were confident in interacting with people in society and asking for help in spite of language problems.

‘…then I asked a kind man who was smiling at me, how can I find the way to my home? Where is ‘torg’ [square, not referring to any specific place]? There was a police car. They took me home. The police were very kind. They are very kind here. If you get lost, they find you and drive you home. Every time, I get lost, I ask people where is the ‘torg’? I live near ‘torg’. They are kind; they take me home.’ (68-year-old female)

-  
*Feeling Isolated*

Some participants complained of their difficulties integrating into the new society because of language difficulties. For example, one of the male participants expressed their longing to be active and get responsibilities in the society like finding a part time job. They also felt they were losing the connection to everyday life or the flow of life when they did not know the language. They expressed their need to feel they are still valued.

## Discussion

Our study provides insights to the life experiences of a group of people who have experienced immigration to another country in addition to experiencing the development of dementia and living with it. The study also reveals that people with dementia are able to discuss their lives and that their views of life has not been erased by dementia as Fazio and Mitchell [25] and Westius, Andersson, and Kallenberg [26] have also pointed out. Our results show that people with dementia no longer feel competent at home according to those who knew their previous performance and that they have lost their earlier positions within their families, a finding similar to Beattie, Daker-White, Gilliard, and Means’ [27] study. Our study also shows that people with dementia feel incompetence that accompany family conflicts, which also were found by Gilmour and Huntington [5] in a New Zealand context and Mazaheri and others [28] in an Iranian context. In spite of feeling incompetent in performing the roles they had in their families, they still felt they were loved and respected by their families. In the public sphere, they found themselves confident and secure when interacting socially. However, some felt they were becoming isolated since they were not fluent in the local language and did not have any responsibilities in society.

Living with dementia meant living with forgetfulness for the Iranian immigrants in Sweden participating in our study. This finding is similar to that found among Iranians living in Iran [28]. Cultural values and understanding have been found to impact the way people assign meaning to dementia and its causes [29]. A study of four ethnic groups in the United States showed that Hispanic and Chinese people believed that dementia was part of a normal ageing and that this belief delays them from contacting healthcare systems for help compared to Anglo Americans [30]. Iranian immigrants in Sweden mainly associated developing dementia to their life difficulties, which was their way of finding a meaning for the disease. It is also interesting that some of the Iranian immigrants in our study regarded forgetfulness as the health problem they themselves suffered from, while they regarded dementia as a disease that people with low IQ or low social class suffered from. They seemed to try to distance themselves from having dementia, to attempt to minimize the effects of forgetfulness, and to rationalise their forgetfulness. It seems that they tried to rebrand their condition of living with dementia, which is a label of a disease, to one of forgetfulness, which may sounds more acceptable to them. Korean Americans [31] and Chinese Americans [32] perceived dementia as a form of insanity. Ayalon and Areán [13] found that Anglo Americans have more accurate knowledge of Alzheimer’s disease than older Latino, Asian, and African American adults. People need a clear explanation of their illness, its nature, and how it develops in order to be able to accept the disease and actively seek help to effectively deal with their dementia-induced situations. Having a distorted understanding of dementia might delay diagnosis and discourage people from seeking treatment [33]. Educating people with dementia as well as their families about the course of the disease, its progress, and its prognosis could help them get a better understanding of it. Public education is also necessary to make people more aware of dementia, as well as complications and consequences for the people with dementia and their families. Such awareness can increase understanding and respect for people with dementia since many behavioural and communication problems can be attributed to the disease and not to their individual personalities.

There was a difference in how the Iranian immigrants in the present study felt about their competence in managing their everyday lives between the private and public spheres, as at home and in their families; they were faced with their failures by people who knew them from before. Those close to them who belonged to the same culture could recognize the inappropriate behaviours of the people with dementia with a reference to their past history. Outside their homes, on the contrary, they felt secure and confident that they could manage things, since no one criticized them or showed their dissatisfaction, at least not in a way the people with dementia recognised. It might be that people in society did not react visibly to their ‘odd behaviours’, as they did not know these people and their level of abilities prior to dementia onset and therefore had other expectations for their behaviour. In encounters with Swedish society, where the participants were not familiar enough with Swedish expressions and cultural codes to the same degree as with their own culture, they interpreted the situation with reference to their understandings of their own culture. As Booker [34] presented, the subjective experience of the person is assumed as the reality, and it should be the starting point in explaining their behaviour and planning support services.

Our finding that people with dementia felt confident and secure to move around in society is significantly different from other published studies on people with dementia in living in their country of origin. Kelly [35] reports that people with dementia experienced ill-being, and Sabat, Napolitano, and Fath [36] report experiencing the embarrassing social identity of a dysfunctional person outside their homes and in society. Even Iranian people with dementia in Iran [28] felt very restricted from going outside and were dependent on others to accompany them in outdoor activities, while participants in our study felt comfortable and secure in performing activities outdoors even though they had experienced getting lost and followed home by police. One possible explanation could partly be the contextual differences. Iran and Sweden are two different contexts with regard to care systems and their impact on the experience of dementia, among other aspects. Iran, with a family-based care system, has high prevalence of intergenerational care and relatively strong filial obligations for taking care of relatives with health problems, including dementia. Such a situation is exactly the opposite of Sweden, where publicly funded care services are available to all across the country and are highly regarded [37]. However, families in Sweden still feel responsible for their parents’ well-being [38]. So the family members of people with dementia in Sweden do not feel embarrassed or a burden to society if they cannot manage all caregiving complications, as much as people in family-based care system. In Iranian communities, there is a high demand on families to provide comprehensive care for their seniors with dementia. Therefore, they restrict the outdoor activities of their relatives with dementia in order to minimize their risk of getting lost, being disturbed by people, or getting hurt in traffic, etc.

A limitation of the study as many other qualitative studies is the small sample size and a one-time interview with each participant. Even though conducting multiple interviews might have been contributed in collecting more information, it decreases the spontaneity of the answers. We do not intend to generalise the findings of this qualitative study; but the findings can be transferred to similar contexts and to people with shared history and characteristics. However, it could be the basis for further quantitative studies.

As with any interview study, there is a likelihood of producing different results with different investigators, for example, by having a non-Iranian interviewer or researcher. However, having an interviewer that knows the culture and was able to communicate in the mother tongue of the interviewees (Persian language) could have helped them to open up and facilitate sharing their experiences. Including other sources of data collection like observation could also have enriched the results.

Our study provides new insights to ways of experiencing dementia by taking into account the immigration experience on the experience of the disease. The insight to the concerns and experiences of people with dementia can help nurses to meet them where they are in their unique contextual situation. Therefore, nurses can help people with dementia obtain a meaningful life with dementia. To get old and develop dementia in a country different than one’s country of origin, in which cultural codes and understanding of situations differ from those in the new homeland, seems to pose a different kind of understanding of living with dementia. Healthcare professional should be sensitive to variations of the experiences of dementia in providing care and support services to them and their families. Being aware of possible differences in how different people experiences living together with dementia can prevent stereotyping in healthcare planning, interventions, and outcome evaluations.

The study contributes in providing a basis for nurses and other healthcare workers to look at the world from the perspective of people with dementia and be sensitive to their life stories and practices, which is a key element to appropriate interventions.

### Methodological considerations

To ensure trustworthiness in our study, the following measures were taken according to Graneheim & Lundman (20) and Lincoln and Guba [39].

To achieve credibility, the authors discussed the different steps of the analysis process back and forth. Some authors had expertise in the field of dementia diseases and some in qualitative content analysis. Having five authors involved in the analysis and discussion contributed to being more cautious about whether the final themes covered the data and decreased the risk of excluding some data because of misidentification as irrelevant data. The researchers tried to be responsive by working inductively, using only data, and not any previously held assumptions, focusing on study data, listening to them, and peer debriefing. To ensure an audit trail as one of the main characteristic of credibility, all the interviews were recorded and transcribed verbatim and the process of developing themes in the analysis was noted. The participants had varied experiences and had different gender, age, and education backgrounds which helped to cover a range of variation in the results. Care was taken to choose the most suitable meaning units, sub-themes, and themes. To show the similarities and differences between sub-themes, representative quotations from the transcribed text were carefully selected. During analysis and drafting the results, agreement among co-authors was sought.

To address transferability of the study, we strived to give a clear description of the participants and process of recruitment to the study, context, and all the steps of the research process, followed by an accurate presentation of the findings together with appropriate quotations. By asking the same area for all interviewees with the same initial question and having an open dialogue within the research team, we attempted to ensure dependability of the study. The results of the study have been shared with other researchers in research seminars in order to discover potential blind spots.

## Conclusions

Living with dementia for the participating Iranian immigrants in Sweden meant living with forgetfulness, feeling incompetent but still loved and feeling confident and secure but also isolated in the society. Since looking at the world from the perspective of the people with dementia is a key element in providing appropriate services for them [40], our study can provide a better basis for healthcare workers to understand the experiences of living with dementia from this specific perspective. People with dementia need to be helped in feeling useful, in making valued contributions to their own and others’ activities. Their families also need to be more aware of the world of people with dementia in order to provide a supportive environment. Educating family members and the people with dementia about the course and process of dementia can help them understand the changes better and adjust their expectations.

## Authors’ information

MM: BScN, RN, MScN, PhD, Senior Lecturer; LEE: RN, MSc, PhD, Associate Professor; ANN: BScN, MScN, PhD, Professor; HS: RN, PhD and KH: MA, PhD.
